# Understanding the influence of online misogyny in schools from the perspective of teachers

**DOI:** 10.1371/journal.pone.0299339

**Published:** 2025-02-26

**Authors:** Harriet Over, Carl Bunce, Jonathan Baggaley, David Zendle

**Affiliations:** 1 University of York, York, United Kingdom; 2 PSHE Association; Technologico de Monterrey, MEXICO

## Abstract

Young people are increasingly exposed to toxic online misogyny through social media. However, to date, it is not clear how exposure to online misogyny might be influencing the behaviour and experiences of adolescents and children. As a first step towards answering this question, we gathered data on how such influences are perceived by surveying 200 teachers, 100 of whom were based in secondary schools (working with children aged 11 and above) and 100 of whom were based in primary schools (working with children aged 4–11). 76% of secondary school teachers and 60% of primary school teachers reported that they were extremely concerned about the influence of online misogyny in their schools. When asked to describe an example of the ways in which online misogyny was influencing the behaviour and experiences of male pupils in their schools, teachers referenced cases in which male pupils praised misogynistic influencers, made misogynistic comments and engaged in discriminatory behaviour towards female peers and staff. When asked to describe an example of the ways in which online misogyny was influencing the behaviour and experiences of female pupils in their schools, teachers referenced cases in which female pupils were the victims of misogynistic behaviour and their well-being was adversely affected. 90% of secondary school teachers and 68% of primary school teachers felt that their school would benefit from dedicated teaching materials to address the impact of online misogynists within their schools. Implications of these data for interventions to combat the rise of online misogyny are discussed.

## Introduction

Extreme misogyny is increasingly finding an audience on social media in the ‘manosphere’, a broad community of individuals which has emerged over the last 15 years. The manosphere incorporates a variety of groups including self-styled pick-up artists, incels (short for involuntary celibates), MGTOW (short for “men going their own way”) and proponents of the red and black pill. Pick-up artists purport to teach men how to seduce women, often advocating the use of deceptive and even coercive tactics [1]. Incels claim to be doomed to a life of singledom often expressing extreme resentment at the women who they imagine deny them sex [2, 3]. MGTOW claim that women, particularly White Western women, are so influenced by feminist ideologies that they should be avoided completely in favour of a life of solitude or a partner from a country with more conservative gender attitudes [4]. Advocates of red and black pill ideologies draw on scenes from the Matrix film franchise in order to explain their relationship to contemporary society. In the Matrix, the protagonist is offered a choice between different coloured pills in order to understand reality or remain in the dark. These groups claim to have taken a pill which has allowed them to see the truth about gender politics, that society is fundamentally biased in favour of women [2]. While red pill and black pill ideologies are closely related, the black pill is typically understood as a more extreme and nihilistic perspective on gender politics [2].

What unites these different groups within the manosphere is a fundamental belief that women are inferior to men and ought to be subordinated to them. Women are often derogated as innately illogical, selfish, greedy and scheming. These attributions are used to justify harassment, coercive control, and discriminatory behaviour [2, 4]. For example, self-styled ‘life coach’ Andrew Tate regularly suggests that women belong in the kitchen and owe their male partners service and gratitude [5]. Other misogynistic influencers, such as Pearl Davis, campaign to restrict voting rights to men [6]. Some influencers within the manosphere explicitly advocate violence against women and appear to have inspired real world attacks against them [3]. These communities are increasingly politicised and often advocate for far-right politicians [7].

Until recently, this international movement has been a hidden problem. Although some influencers have gained widespread notoriety, many others do not engage with mainstream news channels. As a result, the scale of the manosphere may be underestimated by many adults unless they have regular contact with teenagers and young people. Teenagers and young people are regularly exposed to this content through social media and video sharing platforms including Reddit, TikTok and YouTube. While there is relatively little academic research on the extent of exposure, a poll commissioned by the charity Hope not Hate in 2023 found that 80% of 16- and 17-year-old British boys had consumed content created by Andrew Tate. In contrast, only 60% of boys in the same age group had heard of the British Prime Minister [8]. A recent poll by YouGov revealed that 27% of young men in the UK (aged between 18 and 29) hold a positive view of Andrew Tate, and 24% agree with his views about women [9]. A related poll conducted by Internet Matters (2023) suggested that 56% of fathers under the age of 35 approve of Andrew Tate [10].

The dynamics of social media create a context in which misogynistic views are able to flourish. Influencers compete for hits and shares within a limited marketplace of attention. Within this marketplace, controversial and extreme opinions are particularly likely to receive attention, leading to a radicalisation of viewpoints. Algorithms designed to offer users more of the content that they like, combined with systems for blocking unfavourable comments, create echo chambers in which individuals are presented with an increasingly homogenous and polarised world view [11].

To date, there is little evidence relating to how online influencers are affecting the behaviour and experiences of children. As a first step towards answering this question, we conducted a survey with 200 school teachers working in British schools. We surveyed 100 secondary school teachers (working with children aged 11+) and 100 primary school teachers (working with children aged 4–11). We asked participants a series of questions about online misogyny in their schools. We asked them to rate how concerned they were about this problem and to describe the last time they 1) witnessed online misogyny influencing the behaviour or experiences of male pupils in their schools 2) witnessed online misogyny influencing the behaviour of or experiences female pupils in their schools 3) were personally affected by the influence of online misogyny over their pupils. We also asked them what their school was currently doing to tackle the influence of online misogyny and what they thought could be done to tackle this problem within schools. Finally, we asked them to what extent they felt their school would benefit from dedicated teaching materials to tackle the influence of online misogyny over their pupils.

## Method

### Open science and ethical review

This study was pre-registered. The pre-registration details can be found here https://aspredicted.org/blind.php?x=W45_DTB. The survey, coding scheme, anonymised summary data, and second coding are available on OSF: https://osf.io/6udpt/. The study received ethical clearance from the Department of Psychology at the University of York (approval number 2281).

### Participants

Participants were recruited through the online testing platform Prolific and were pre-screened to ensure that they were working as teachers in British schools. The survey itself was built with Qualtrics. All participants provided their written informed consent.

100 participants (61 female, 38 male, 1 non-binary, mean age 39.7, age range = 22–64) worked at British secondary schools (accepting children aged 11–16 or 11–18). Of these teachers, 86 reported that they worked in state-sponsored schools and 14 reported that they worked in fee paying schools. 94 of these participants worked in schools with a mixed gender intake accepting male and female pupils, 2 worked in single-sex boys schools and 4 worked in single-sex girls schools. Participants reported that they taught a range of subjects including mathematics, science, English, computing, sport, history, geography and PSHE (personal, social, health, and economic education).

100 participants (80 female, 20 male, mean age = 38.5, age range = 21–62) worked at British primary schools (reporting that they taught children aged 4–11 years). Of these teachers, 91 reported that they worked in state-sponsored schools, 8 reported that they worked in fee paying schools and 1 did not specify. 99 of these teachers worked in schools with a mixed intake accepting male and female pupils, and 1 worked in a boys’ school.

A further 29 participants were tested but excluded because they worked in schools that taught primary and secondary school aged pupils (12). We excluded these participants because we could not be sure whether the examples they raised were drawn from primary-aged children or secondary-aged children. We also excluded teachers who reported that they worked in early years settings with children under the age of 4 (4) or because they worked in a further or higher education setting with children older than 16 (12). 1 participant was excluded because they did not specify what type of school they worked in.

Participants were reimbursed £2 for taking part. Participation took, on average, 8 minutes and 16 seconds (*SD* = 7 minutes and 36 seconds). Secondary school teachers typically took somewhat longer to complete the survey. On average, secondary school teachers took 10 mins 0 seconds over their answers (SD = 9 mins 28 seconds) and primary school teachers took 6 mins 33 seconds (SD = 4 mins 30 seconds) over their answers. Data collection took place between 3^rd^ November 2023 and 8^th^ November 2023.

### Design

#### Understanding the influence of misogyny in schools

Our principal goal was to understand the extent to which teachers felt that online misogyny was influencing pupils in their schools. In order to address this question, we asked teachers one overarching question and three more specific questions. As an overarching question, we asked them the extent to which they agreed with the following statement: “I am extremely concerned about the influence of online misogyny on pupils in my school”. Answers were collected on a four-point likert scale: 1 (not at all) 2 (a little bit) 3 (moderately) 4 (strongly).

In order to understand the ways in which online misogyny might be affecting male pupils, we asked teachers “Can you tell us about the last time that you observed online misogyny affecting the behaviour or experiences of a male pupil in your school?”. In order to understand the ways in which online misogyny might be affecting female pupils we asked “Can you tell us about the last time that you observed online misogyny affecting the behaviour or experiences of a female pupil in your school?”. Finally, in order to understand the extent to which teachers might be affected by the influence of online misogyny over their pupils, we asked “Can you tell us about the last time that you were personally affected by the influence of online misogyny on your pupils?”.

#### Understanding current practice and what strategies might be effective in addressing misogyny in schools

As a first step towards understanding current practice in how schools address any issues raised by online misogyny we asked teachers “What, if anything, is your school currently doing to tackle the influence of online misogynists?”. In asking this question, we sought to understand whether there are gaps in the current provision that might usefully to be addressed in future research and practice.

In order to capture elements of best practice and consider possible avenues for future intervention, we also asked teachers “What sort of strategies do you think might be helpful in addressing the influence of online misogynists in schools?”

Finally, we asked teachers the extent to which they agreed with the following statement “My school would benefit from receiving dedicated teaching materials to tackle the influence of online misogyny”. Responses to this question were requested on a four-point likert scale: 1 (not at all) 2 (a little bit) 3 (moderately) 4 (strongly). We asked this question in order to understand whether there is an appetite for school-based interventions focused specifically on tackling online misogyny.

#### Procedure

Participants were asked a small number of preliminary questions relating to themselves and the type of school they worked in. Following this, they were asked the questions outlined above in a fixed order. Finally, participants were asked if they would be interested in participating in further research on online misogyny and whether there was anything else they would like to share with us on this topic. Participants were thanked for their participation, debriefed, and redirected to Prolific for payment.

#### Coding

The coding scheme was developed by the first author who read participants responses multiple times in order to derive relevant themes. Coding categories were not mutually exclusive and teachers’ responses could be coded into multiple categories. In order to examine the reliability of the coding we asked a second rater to independently code 25% of these responses. Agreement between the two coders ranged from .71 to .8 for the different questions. The average Kappa = .75. We adopted an iterative approach to developing a maximally reliable coding scheme. We clarified the coding scheme, increasing its specificity, and asked a third rater to code 25% of the data. Agreement between two coders using this improved coding scheme ranged between .815 and .898. The average Kappa = .862.

For the question “Can you tell us about the last time that you observed online misogyny affecting the behaviour or experiences of a male pupil in your school?”, the first rater coded responses into 6 categories. This rater assigned 59 codes in total. The two raters agreed in 91.4% of cases, Cohen’s Kappa = .891. For the question “Can you tell us about the last time that you observed online misogyny affecting the behaviour or experiences of a female pupil in your school?”, the first rater coded responses into 5 categories and assigned 56 codes in total. The two raters agreed in 89% of cases, Cohen’s Kappa = .845. For the question “Can you tell us about the last time that you were personally affected by the influence of online misogyny on your pupils?”, the first rater coded responses into 5 categories and assigned 58 codes in total. The two raters agreed in 89.7% of cases, Cohen’s Kappa = .863. For the question relating to what schools are currently doing to combat the influence of online misogyny, the first rater coded responses into 11 categories and assigned 68 codes in total. The two raters agreed in 89.7% of cases, Cohen’s Kappa = .898. Finally, for the question relating to what schools ought to do to combat the influence of online misogyny, the first rater coded responses into 13 categories and assigned 61 codes in total. The two raters agreed in 83.6% of cases, Cohen’s Kappa = .815. The total number of codes varied between questions because any given answer from a participant could reference more than one idea or suggestion and so be assigned more than one code. The results reported below are based on the coding by the first rater.

## Results

### Understanding the extent to which online misogyny is a problem in schools

#### To what extent do you agree with the following statement? “I am extremely concerned about the influence of online misogyny on pupils in my school”

**Fig 1:**
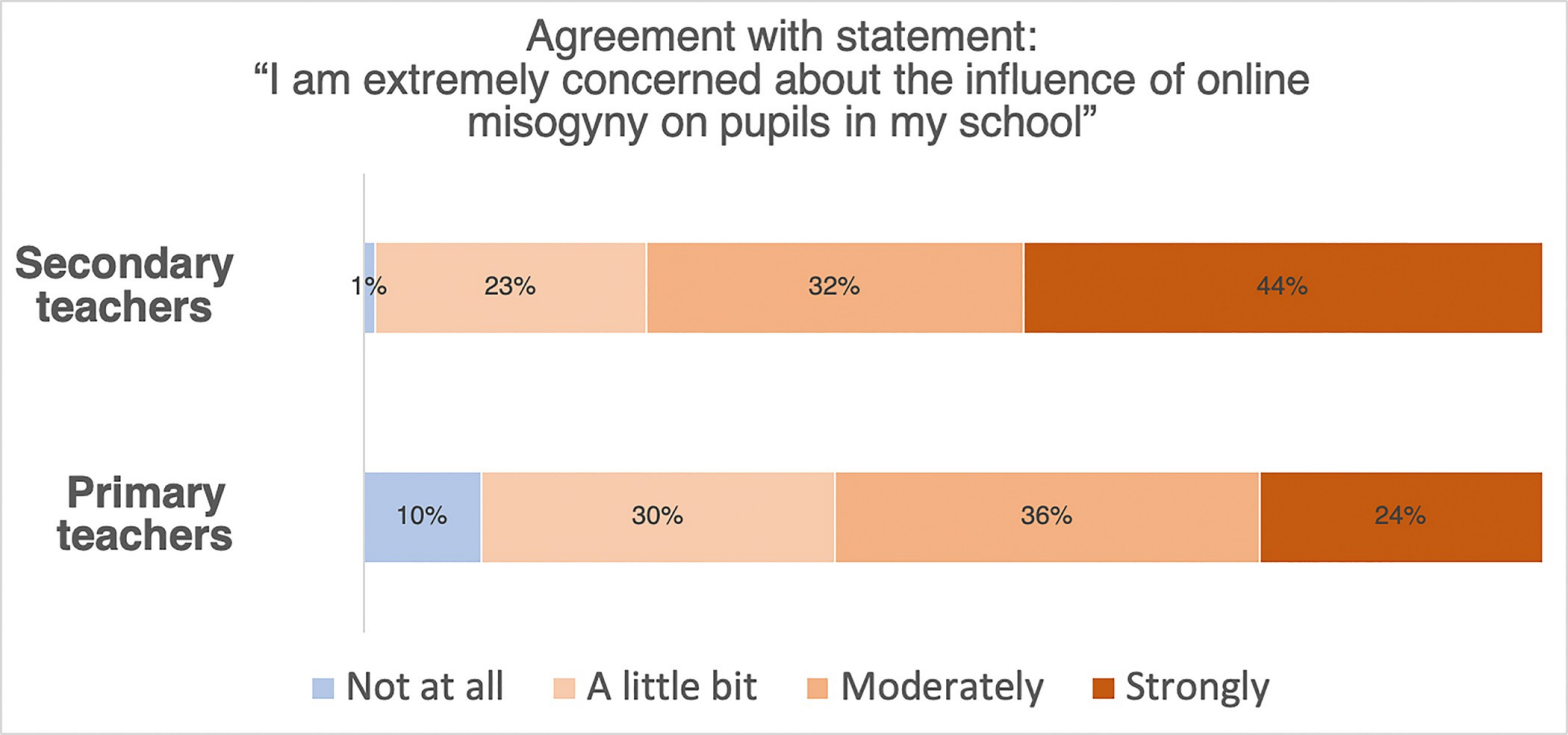
The extent to which secondary school teachers (above) and primary school teachers (below) teachers agreed with the statement “I am extremely concerned about the influence of online misogyny on pupils in my school”.

76% of secondary teachers moderately or strongly agreed that the influence of online misogyny over their pupils was extremely concerning (see Fig 1). These results broadly accord with previous data suggesting that a considerable percentage of British teenagers are exposed to misogyny (Hope not Hate, 2023) and go beyond them by suggesting that this exposure may be negatively impacting pupils in schools. Strikingly, 60% of primary teachers also moderately or strongly agreed that the influence of online misogyny over their pupils was extremely concerning (see Fig 1). These results suggest that dialogues surrounding the influence of online misogyny need to incorporate discussions about primary-aged children as well as adolescents.

In order to understand whether online misogyny is a greater concern among secondary school teachers than it is among primary school teachers, we converted participants’ agreement with the statement into numerical estimates (not at all: 1, a little bit: 2, moderately: 3, strongly: 4). We then subjected these scores to a between subjects t test. This analysis suggests that secondary school teachers are significantly more concerned than are primary school teachers (*t*(198) = 3.60, *p* < .001).

#### Can you tell us about the last time that you observed online misogyny affecting the behaviour or experiences of a male pupil in your school?

When asked to describe a recent situation in which they observed online misogyny influencing the behaviour or experiences of male pupils in their school, teachers tended to reference instances in which male pupils made misogynistic comments and engaged in discriminatory behaviour.

#### Secondary school teachers

As 4 teachers reported that they worked in single sex girls’ schools, the following percentages are based on 96 responses and rounded to the closest percentage point. 38% of secondary school teachers referenced male pupils making misogynistic comments, 14% referenced male pupils engaging in discriminatory or inappropriate behaviour and 14% of teachers referenced male pupils disrespecting female members of staff relative to male members of staff. 26% of teachers referenced male pupils discussing misogynistic influencers like Andrew Tate or misogynistic movements from the internet such as incels. Example responses from teachers elaborating on the ways in which online misogyny appears to be influencing the behaviour and experiences of their male pupils are presented in Table 1.

**Table 1:** Responses given by secondary school teachers and primary school teachers when asked to describe the last time they witnessed online misogyny influencing the behaviour or experiences of male pupils in their school. Responses marked with a * were coded into more than one category. In all tables, spelling, punctuation and grammar are retained from the original responses.

School type	Coding category	Example responses
Secondary	Derogatory comments	In the past year, I have observed a pupil discussing how it would not be rape if nobody found out. It is believed this stemmed from online experiences.
		A male student making comments about suitable jobs for girls and boys in an employability session. He had seen a video of Andrew Tate talking about what was ’high value’ and took the view that men should be in positions of power, describing how girls would not be good leaders.*
		The last time, a student made derogatory comments towards another student who was female, when she challenged him he told her that women would soon not be allowed outside and that we’d be back "where we belong", he made several references to women being "too big for their boots" and how we make things up. When I asked where he got these opinions he told me he liked to watch Andrew Tate.*
	Discriminatory behaviour	Several male pupils with girlfriends who are abusive towards them in every aspect: physically, emotionally.
		A male pupil was reported to the school for messaging female pupils in an extremely inappropriate sexualised manner using some of the discourse often associated with the likes of Andrew Tate*.
		They try to physically intimidate females with their presence. They are well versed in the online world having access to Youtube and other online content.
	Disrespecting female staff	One pupil sent a number of sexist and harassing messages to a young female member of staff. During the aftermath it became clear that he was strongly influenced by Tate et al. *
		There have been several instances of male pupils not respecting female members of staff, not reacting well to instruction by female members of staff or being heard to make derogatory statements about the looks of female members of staff.
		Very recently—Male pupil, who was open about having accessed Andrew Tate’s material, made unacceptable, sexist comments about a female teacher when she gave him a consequence.*
	Discussing misogynistic influencers	Yes, certain pupils were ingesting online content from so called "Incels" and in the Incel movement. As a result, various derogatory jokes were made for those males that are not so well off or come from poorer families.
		A group of KS4 boys were gathering in the corridor shouting ’free Andrew Tate.’
		Recently I heard hero worship Andrew Tate and think his ways were gospel. They talk about sharing his videos.
Primary	Derogatory comments	A male pupil told me it was ’ok to hurt women because Andrew Tate does it’*
		I once saw a boy say to a girl that she belonged in the kitchen and he was a very young age. It was a little bit shocking.
		A student once reiterated something he learned from online misogynists such as Andrew Tate. They were role-playing different careers and he had a genuine belief that the girls were not allowed to play with them because they shouldn’t have careers as females.*
	Discriminatory behaviour	I have noticed children talking about people such as Andrew Tate in the playground. They are then not as friendly with the girls in their class.*
		Boys touching girls non consensually and not understanding why they couldn’t.
		A few months ago I had a group of male students referring to themselves at the Top Gs and excluding female students from activities*
	Disrespecting female staff	Children mention Andrew Tate a lot and laugh about degrading women. A boy has said he doesn’t respect female teachers*
		Certain males will not listen to female teachers and says ’I don’t need to listen to you’.
		Bad attitude towards female members of staff but not towards male staff
	Discussing misogynistic influencers	Students were asked to think about a role model in their life, more than one boy wrote Andrew Tate. One boy specifically began to discuss how cool Andrew Tate is and why he is so inspiring.
		There are regular mentions of figures such as Andrew Tate, and mimicking language and behaviour that students have seen online from influencers such as him.

Only 11% of teachers reported that they had not witnessed online misogyny influencing the behaviour or experiences of their male pupils. 16% of teachers gave responses that were too vague to classify for example ‘I am aware of this’ or ‘I handled a case involving two male pupils’.

#### Primary school teachers

23% of primary school teachers referenced their male pupils making misogynistic comments. 12% of primary school teachers referenced their male pupils engaging in discriminatory or inappropriate behaviour and 8% referenced male pupils disrespecting female members of staff relative to male members of staff. 17% of primary school teachers referenced male pupils discussing misogynistic online influencers. Example responses outlining specific examples from each category can be seen in Table 1.

37% of primary school teachers reported that they had not observed any influence of online misogyny over the behaviour or experiences of their male pupils. 13% of primary school teachers gave responses that were too vague to place into these categories.

#### Can you tell us about the last time that you observed online misogyny affecting the behaviour or experiences of a female pupil in your school?

When asked to describe a recent situation in which they observed online misogyny influencing the behaviour or experiences of female pupils in their school, teachers tended to reference ways in which girls were the victims, rather than perpetrators, of discriminatory or inappropriate behaviour.

#### Secondary school teachers

Two participants worked in single sex boys’ schools, so the following percentages are based on 98 responses. As above, reported percentages are rounded to the nearest whole number. 44% of secondary school teachers referenced female pupils being the victims of misogynistic comments, discriminatory, or inappropriate behaviour directed at them by other pupils. 30% explicitly referenced the negative impact of other pupils’ behaviour on their well-being, self-esteem and engagement. Only 1 teacher referenced a female pupil talking about a misogynistic influencer (Andrew Tate) in a positive way. See Table 2 for example responses illustrating the ways in which teachers feel that online misogyny is influencing their female pupils.

**Table 2:** Example responses given by secondary school teachers and primary school teachers when asked to describe the last time they witnessed online misogyny influencing the behaviour or experiences of female pupils in their school. Responses marked with a * were coded into more than one category.

School type	Coding category	Example responses
Secondary	Victims of inappropriate behaviour	There have been a few instances of female pupils being called unkind names based on their looks/appearance which seem to have been related to online misogyny. For example, comments such as "you’d not make much money on Only Fans" or "I wouldn’t even rape you" and the like which are overly sexual and highly demeaning.
		The incel movement resulted in various pupils criticising female pupils who had partners before in a very insulting manner.
		Girls spoke to me about the problem of `upskirting’ on the stairs
		Recently, this girl (one of my pupils) was attacked by several boys, who told her that her looks were awful and that she also looked dense.
	Negative impact on well-being	Female students are humiliated and angry by it. They are frustrated and lose their temper sometimes
		Upsetting the female pupil, resenting school, not wanting to attend
		A female pupil very upset by an online video she had been sent by another male pupil, which was extremely misogynistic*
Primary	Victims of inappropriate behaviour	A student once reiterated something he learned from online misogynists such as Andrew Tate. They were role-playing different careers and he had told a female pupil to “go back to the kitchen”because “women aren’t allowed to have jobs”.
		Girls are often subjected to unpleasant behaviour from groups of boys who are copying what they have seen online.
		A number of pupils have had issues with online misogyny, including predatory behaviour towards some of the girls in my class.
	Negative impact on well-being	The majority of the girls in my class have been worried about coming to school due to what the boys may say or do to them.
		I had a female pupil complain last week of an online porn group that a group of boys were adding her into and telling her it was how she should be. The effects were negative for her.*
		Boys in my class used to regularly quote Andrew Tate and reference his videos, to the disgust of many of the girls.

Only 30% of teachers could not describe an incident in which online misogyny had impacted female pupils. 11% of teachers gave responses that were too vague to classify into these categories, for example responding ‘every day’ or ‘all the time’ without specifying the nature of the impact further.

#### Primary school teachers

As 1 participant worked in single sex boys’ schools, the following percentages are based on 99 responses and reported percentages are rounded to the nearest whole number. 40% of primary school teachers referenced female pupils being the victims of misogynistic comments, discriminatory or inappropriate behaviour directed at them by other pupils and 16% explicitly referenced the negative impact of other pupils’ behaviour on female pupils’ well-being, self-esteem and engagement. One primary school teachers referenced female pupils discussing misogynistic influencers but it was not clear whether or not this was in a positive way. See Table 2 for example responses.

Close to half of primary school teachers (49%) could not describe a recent incident in which online misogyny had impacted female pupils. 6% of teachers gave responses that were too vague to classify into these categories.

#### Can you tell us about the last time that you were personally affected by the influence of online misogyny on your pupils?

Teachers varied in how they interpreted this question. Whereas some teachers referenced times where they had witnessed and/or challenged inappropriate comments and behaviour from their pupils, other teachers described the influence on their own well-being, time, and capacity to teach. Echoing responses from previous questions, some teachers referenced being disrespected by their pupils and receiving sexist abuse from pupils.

#### Secondary school

48% of secondary school teachers referenced occasions on which they had witnessed and/or challenged misogynistic comments or behaviour among their pupils, 22% referenced the impact that addressing online misogyny had over their well-being, time or capacity to teach. 19% of secondary school teachers referenced being disrespected by their pupils. All but two of the teachers who mentioned being disrespected by their pupils identified as female. See Table 3 for example responses illustrating the ways in which teachers feel they are personally affected by the influence of online misogyny over their pupils.

**Table 3:** Example responses given by secondary school teachers and primary school teachers when asked to describe the last time they were personally affected by the influence of online misogyny over their pupils.

School type	Coding category	Example responses
Secondary	Witnessing and challenging inappropriate behaviour	On a weekly basis I am called to try and sort out that type of situation. They happen out of schools but the consequences reach the classroom. Most common is girls being sexualized and called sluts or other derogatory terms.
		I’m just left gobsmacked by the attitude, they also don’t believe that what they say or how they act is problematic, which is the problem!
	Negative impact on well-being	I am always affected by any issue which I deal with when unacceptable comments which have been made, usually after watching misogynistic material online. It causes great upset to the victim and it is frustrating to see previously pleasant male pupil’s being brainwashed.
		As a gay person I am very aware of the language used—the word "GAY" is often used to denote negative qualities. I also feel sometimes an underlying threat that if I discipline a boy at the school he could turn around and claim I had touched him inappropriately etc
		It can be quite demoralising to see the behaviour of some students that can get so affected by what they watch online, even if you spoken to them about what they’re watching, and the implications of this.
	Being disrespected by pupils	Students have made comments that my role as a teacher was not important as I was a women, and that I should be at home instead. I have had pupils tell me that they do not respect me as I am a woman and do not need to listen to things I say.
		Yes, my male students have no respect for female teachers. They would not respond to any of my requests and only quieted down when a male teacher was present. They are well aware of famous male figures online with misogynistic views and I feel that they try to emulate their behaviours at school
		A couple of boys have laughed at me when I have given instructions and ignored me followed by comments such as “Why don’t you shut up and make me a sandwich”.
Primary	Witnessing and challenging inappropriate behaviour	Students regularly come to me and complain about the way they are spoken about by others in the school setting. For example, one student was labelled a ’whore’ on a whatsapp group.
		The girls were very upset after reading comments from boys that were degrading and belittling them
	Negative impact on well-being	All of this effects us as teachers. We take on these troubles and try and protect other people’s children from it.
		It affects me daily as I am having to deal with incidents most days that are a result of this behaviour.
		Just in dealing with the fallout between pupils and spending a lot of time on it rather than teaching.
	Being disrespected by pupils	I would say the child that actively ignores female teachers is extremely hard. There is no respect to his female peers either.
		Some boys have less respect for female teachers and can need to see you asserting authority and creating a boundary. This is quite common.

Only 25% of secondary school teachers reported that the influence of online misogyny over their pupils did not affect them personally. 6% gave responses that were too vague to classify for example saying things like “during discussion in class” or “last week” without specifying further.

#### Primary school

24% of primary school teachers referenced occasions on which they had witnessed and/or challenged misogynistic comments or behaviour among their pupils and 10% referenced the impact that addressing online misogyny had over their well-being, time or capacity to teach. 8% of primary school teachers referenced being disrespected by their pupils. All of the primary school teachers who mentioned being disrespected by their pupils identified as female. See Table 3 for example responses.

An overall majority of primary school teachers (61%) reported that the influence of online misogyny over their pupils did not affect them personally. 4% of primary school teachers gave responses that were too vague to classify.

#### What can and should be done to tackle the influence of online misogyny in schools?

*What*, *if anything*, *is your school currently doing to tackle the influence of online misogyny on pupils*? ***Secondary school teachers*.** A minority of teachers reported that their school was directly tackling the influence of online misogyny over their pupils. 30% of teachers reported that their school sought to teach their pupils about stereotyping, prejudice and discrimination more broadly or about misogyny specifically. Among those teachers who referenced teaching their pupils about misogyny, several mentioned specific content related to misogynistic influencers like Andrew Tate. It thus appears that, despite serious concerns about the ways in which online misogyny is influencing the behaviour and experiences of secondary school pupils, dedicated classes seeking to help children critically reflect on misogynistic content are relatively rare.

Other teachers reported that their school engaged their pupils with conceptually related topics that could plausibly help mitigate the influence of misogynistic influencers. 11% of teachers referenced classes designed to teach pupils values such as respect, empathy, and fairness. 4% of teachers reported that their school sought to highlight positive male and/or female role models for their pupils. 15% of teachers referenced classes designed to teach pupils how to stay safe online including classes about misinformation.

Rather than referencing the content of classes and conversations with pupils, other teachers referenced discipline. 15% of teachers described the ways in which their school addressed the behaviour of pupils who used misogynistic language or acted in a misogynistic way. Whereas some teachers reported that their school focused on strict discipline and a ‘zero-tolerance’ policy, other teachers referenced the concept of ‘restorative justice’ and engaging in dialogue with students who expressed problematic views.

A small minority of teachers (8%) referenced that staff had received training related to online misogyny. This training included team discussions, workshops on online misogyny, and the distribution of information about online influencers such as Andrew Tate to staff.

8% of teachers reported that their school sought to communicate with parents about online misogyny. Some teachers reported that their schools communicated with all parents about this issue, for example in the form of regular bulletins. Other teachers reported that their school communicated with parents when their children engaged in problematic behaviour. 4% of teachers reported that their school limited access to the internet in school in part to limit engagement with online misogyny.

11% of teachers reported that they were not aware of any action their school was taking to combat the influence of online misogyny on their pupils.

Finally, 18% of teachers gave responses that were too vague to classify into any of these coding categories. For example, simply saying “in assemblies” or “running lessons to highlight these issues” without specifying further.

***Primary school teachers*.** A minority of primary school teachers, 14%, reported that their school taught their pupils about discrimination and/or sought to tackle the influence of online misogyny specifically.

Some teachers referenced teaching related topics that could plausibly be a protective influence against online misogyny in a broad sense. 23% of teachers referenced that their school sought to teach their pupils positive values such as empathy, fairness and respect. 4% of teachers referenced that their school sought to highlight positive role models. Whereas some teachers emphasised the importance of male role models, others emphasised the importance of female role models. The most common response among primary school teachers related to teaching pupils how to stay safe online, which was mentioned by 40% of participants.

Rather than focusing exclusively on the content of classes and conversations with pupils, some teachers referenced discipline. 9% of primary school teachers referenced tackling the behaviour of pupils who made misogynistic comments or acted in a misogynistic way, 4% referenced limiting pupils’ access to the internet as a way of tackling online misogyny and 3% referenced communicating with parents as a way of tackling online misogyny.

18% of primary school teachers were not aware of any action their school was taking to tackle the influence of online misogyny on their pupils. Finally, 11% of responses were too vague to classify into a coding category.

*What sort of strategies do you think might be helpful in addressing the influence of online misogynists in schools*? Teachers offered a range of suggestions for how the influence of online misogyny could be tackled in schools. Answers to this question were typically brief, rarely going into detail about specific content that could be taught in classes or specific policies that would be beneficial.

***Secondary school teachers*.** Among secondary school teachers, 29% referenced the importance of teaching children about discrimination and/or the dangers of online misogyny directly, 12% emphasised the importance of teaching pupils positive values such as fairness and respect. 13% suggested it would be valuable to highlight positive male and/or female role models, 5% referenced the importance of teaching children how to stay safe online, and 5% referenced the value of emphasising the negative consequences of misogyny for victims.

11% of secondary school teachers focused on the importance of discipline, 3% referenced supporting students so they felt comfortable disclosing problems and 3% referenced limiting pupils’ access to the internet. 6% referenced the importance of communicating with parents and 6% referenced the need for staff training. 8% emphasised the need for governmental action.

28% gave responses that were too vague to classify into these categories and 1% of participants did not make any suggestions.

***Primary school teachers*.** Among primary school teachers, 22% referenced the importance of teaching pupils about discrimination and/or online misogyny specifically. 19% highlighted the importance of teaching positive values such as fairness, 10% emphasised the importance of highlighting the negative consequences of misogyny for victims, 10% highlighted the importance of emphasising male and/or female role models and 13% emphasised the importance of teaching children how to stay safe on line.

5% wrote about the importance of discipline and 4% wrote about the importance of supporting students. 9% wrote about the need for communication with parents, 5% suggested that staff training could be beneficial and 1% referenced the importance of governmental action.

22% of primary school teachers gave suggestions to vague to classify and 1% did not make any suggestion.

**Fig 2:**
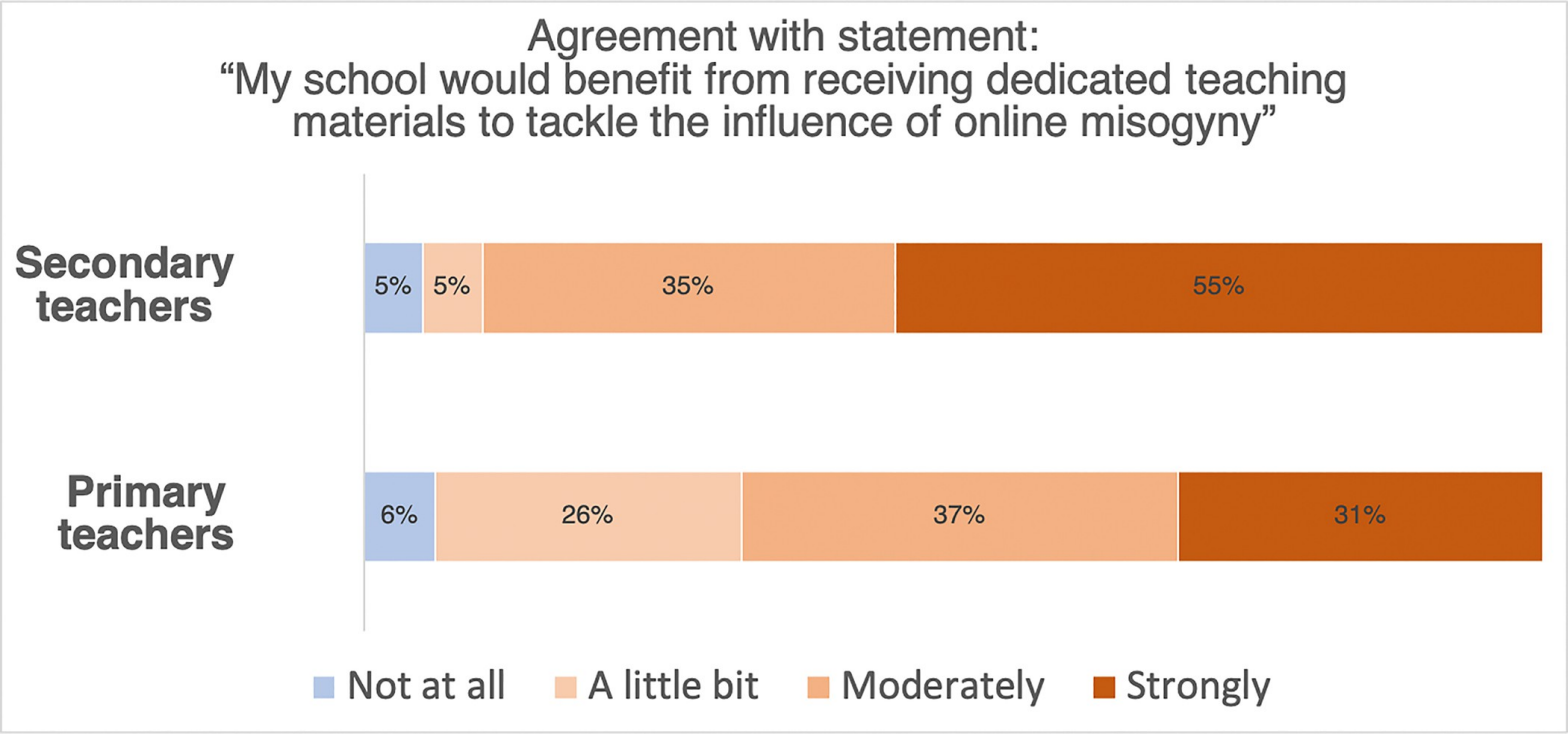
The extent to which secondary school teachers (above) and primary school teachers (below) agree with the statement “My school would benefit from receiving dedicated teaching materials to tackle the influence of online misogyny”.

*Would schools benefit from further resources to help tackle the influence of online misogyny over their pupils*? The overwhelming majority of secondary school teachers (90%) expressed moderate or strong agreement with the statement “My school would benefit from receiving dedicated teaching materials to tackle the influence of online misogyny” (see Fig 2). An overall majority of primary school teachers (68%) also expressed moderate or strong agreement with this statement. A small minority, 5% of secondary school teachers and 6% of primary school teachers, did not agree at all with this statement.

Taken together, our data suggest that online misogyny is associated with substantial problems in schools and that teachers would appreciate assistance in addressing the issues it raises.

## Discussion

There is increasing concern about young people’s exposure to the manosphere [2, 3, 12]. Polling data reveals that a substantial proportion of teenage boys have consumed content created by misogynistic influencers [8]. However, to date it is unclear how interaction with this toxic online environment might be influencing the behaviour and experiences of young people. As a first step towards answering this question, we conducted a survey with teachers.

### Contribution to knowledge

76% of secondary school teachers reported that they were extremely concerned about the influence of online misogynists over their pupils. When asked to give an example of how they felt online misogynists were influencing the behaviour and experiencing of pupils in their school, secondary school teachers referenced male pupils making misogynistic comments and engaging in discriminatory behaviour towards female staff and students. One teacher reported an incident in which a male pupil said “It wouldn’t be rape if nobody found out”. Another teacher reported an incident in which a male pupil said that “women are too big for their boots” and that soon “women wouldn’t be allowed outside”. Other teachers reported cases in which male pupils were abusive towards female partners or sought to physically intimidate them. Some teachers expanded on the negative impact this had on girls’ well-being, self-esteem and/or school engagement. This aligns with previous research suggesting that experiencing sexism has negative implications for women and girls’ well-being and mental health [13].

The majority of public discourse and academic debate, as well as polling data, has focused on the ways in which online misogyny might be influencing the behaviour and experiences of young adults and adolescents [9, 12]. This focus is understandable as children younger than 12 or 13 are not supposed to have social media accounts [14]. Furthermore, influencers themselves appear to target adolescents. Iggy Semmelweis, a crucial organiser behind Andrew Tate’s online platform The Real World, posted that the main demographic for their social media machine was 12- to 18-year-old boys [15]. Nevertheless, it is important to consider the possibility that children younger than 11 may also be influenced by online misogynists. Many children younger than 12 have access to social media and video sharing platforms either directly or through interaction with older siblings, peers, or adults [14].

Our data suggest that online misogyny may be generating social problems among primary school-aged children as well as adolescents. 60% of the primary school teachers we sampled reported that they were extremely concerned about the influence of online misogyny over their pupils. While primary school teachers report being less concerned than do secondary school teachers, these results remain striking.

When asked how they felt online misogynists were influencing the behaviour and experiences of their pupils, primary school teachers also wrote about their male pupils making misogynistic comments and engaging in discriminatory behaviour towards female pupils and staff. For example, one primary school teacher reported an incident in which a male pupil said that it is “ok to hurt women because Andrew Tate does it’”, while another reported a male primary school pupil telling a female pupil that “she belonged in the kitchen”. Another teacher reported that their male pupils “touch girls non-consensually” and “do not understand why this is inappropriate”. Some primary school teachers also expanded upon the impact this had on female pupils. For example, one teacher wrote that “the majority of the girls in my class have been worried about coming to school due to what the boys may say or do to them”. These results suggest that future intervention efforts need to consider action among primary school-aged children as well as secondary school-aged children.

In addition to asking about how online misogyny was influencing the behaviour and experiences of pupils, we also asked teachers what, if anything, their schools were currently doing to combat the influence of online misogyny and what they thought their schools ought to do. Approaches varied widely across schools. Among the secondary school teachers we surveyed, 30% reported that their school explicitly taught its pupils about online misogyny or discrimination more broadly. Among primary school teachers we surveyed, 14% reported that their school explicitly taught its pupils about online misogyny or discrimination. Other teachers reported that their school taught related content that could plausibly help pupils to critically engage with the manosphere, for example offering classes on online safety or teaching positive values such as empathy, respect and kindness. It would be valuable for future to research to systematically investigate the prevalence of sexist behaviours among school pupils and how it varies with the particular approach to teaching discrimination adopted by schools.

The teachers we surveyed suggested a range of possible approaches to tackling online misogyny. These included talking directly to children about misogyny and discrimination as well as focusing on related topics such as online safety and empathy. Other teachers referenced further strategies that could be incorporated into whole school approaches including limiting pupils’ access to smart phones/the internet, communicating with parents and creating an atmosphere in which children feel comfortable disclosing experiences of discrimination. While teachers’ responses to this question rarely contained extensive details, they may nevertheless help inform the development of interventions that can be empirically tested in future research.

Research on anti-bias education suggests that it is crucial to discuss discrimination and its negative consequences with children [16]. Multiple interventions to tackle sexism in schools have been developed by researchers including programmes designed to ensure female representation across the curriculum and encourage girls into STEM subjects [17, 18]. It may be valuable to supplement the most effective among these approaches with a more specific focus on the discriminatory messaging of the manosphere. Indeed, 90% of secondary school teachers and 68% of primary school teachers reported that they felt their school would benefit from dedicated teaching materials on online misogyny.

Taken together, these data may help us to understand female experiences in school. There is growing recognition that poor behaviour in schools is disproportionately affecting female pupils and female teachers. Studies from regulatory bodies, charitable organisations, and academics suggest that sexist harassment of girls is on the rise and is becoming normalised among school-aged boys [19]. In a 2021 Ofsted review of sexual abuse and harassment in schools and colleges in the UK, 92% of girls reported that they or their peers had been victims of sexist name-calling on a frequent basis. Nearly 90% reported that they had received unsolicited and unwanted explicit sexual images [19].

### Study limitations

It is important to acknowledge, however, that our data do not allow for strong causal inference. We asked teachers to tell us about the ways in which they felt online misogynists were influencing the behaviour and experiences of their pupils. However, the causal relationships between viewing online misogyny and holding misogynistic attitudes often remains opaque. Any given example of sexist behaviour, for example disrespecting a female teacher, may be the product of multiple influences including conversations with parents and peers. However, it is noteworthy that some teachers reported that pupils directly discuss misogynistic influencers and use the names of these influencers to justify harassment or derogation of women. For example, one teacher reported that they had “heard hero worship of Andrew Tate” and another reported that “there are regular mentions of figures such as Andrew Tate, and mimicking language and behaviour that students have seen online from influencers such as him”. Other pupils directly refer to social media sites and video sharing platforms when engaging in discriminatory and/or disrespectful behaviour. For example, one teacher described boys telling girls “You wouldn’t make much money on Only Fans”. These responses strongly suggest that online misogyny is one important factor to consider when researching the causal origin of sexist beliefs and behaviours.

When seeking to conceptualise the causal relationship between viewing online misogyny and engaging in sexist behaviour, there are multiple possible pathways. One possibility is that the more online misogyny male pupils view, the more misogynistic their attitudes and behaviour become. This account accords with social learning views of prejudice more broadly [20] and with literature from computer science suggesting that individual engagement with the manosphere appears to become more extreme over time [7, 21]. Another possibility is that individuals already inclined towards misogynistic viewpoints are drawn to the manosphere. Rather than leading to an increase in misogynistic attitudes, misogynistic influencers may offer young people a new means by which to express their discriminatory views. These proposed processes are not mutually exclusive and could, in principle, occur in parallel. Future longitudinal research, measuring changes in pupils’ sexist behaviours and engagement with online misogyny over time, could help shed light on the complex causal relationships in this domain. Both possible causal relationships are a cause of concern–whether online misogyny is causing increases in sexism or licensing its expression, it is a problematic social trend.

It is important to acknowledge other limitations of our research. As we surveyed teachers rather than children themselves, our findings do not allow us to speak to the question of prevalence. We cannot conclude from our data how many children are engaging with misogynistic influencers nor can we ascertain how frequently they are doing so. Furthermore, we cannot infer the age at which, on average, misogynistic influencers first exert an influence over the behaviour and experiences of children, nor whether engagement is changing over time. While polling data has provided some insights into exposure across age, it is crucial to develop more sophisticated methods that allow us to directly assess what content young people are interacting with online over time.

### Future research

In the responses teachers gave to our questions, they tended to focus on the ways in which male pupils who engage with online misogyny perpetrate harm against others. However, it will also be important for future research to consider the ways in which online misogyny may harm boys themselves. One rarely mentioned but important theme from the qualitative data we collected is the ways in which online misogyny might increase discrimination against members of the LGBT+ community, including against gay men and boys. For example, one male teacher reported that “as a gay person I am very aware of the language used—the word "GAY" is often used to denote negative qualities. I also feel sometimes an underlying threat that if I discipline a boy at the school he could turn around and claim I had touched him inappropriately”. The impact of the manosphere on LGBT+ pupils and staff is a crucially important topic for future research.

Another important direction for future research is the extent to which online misogyny may be influencing educational attainment in boys. Our data suggest that male pupils who engage with misogyny sometimes question the authority of female teachers. More broadly, misogynistic influencers often seek to undermine support for traditional systems of authority and academic achievement, instead advocating get rich quick schemes sometimes involving sex trafficking, gambling and cryptocurrency [5]. Previous research has demonstrated that girls outperform boys academically throughout the school years [22]. It is important for future research to assess whether engagement with the manosphere is exacerbating this trend over time.

Perhaps the most important direction for future research is to understand how to tackle the influence of online misogyny over children and young people. Clearly this social problem requires intervention at multiple levels. One aspect of this coordinated approach is lessons designed to help children critically engage with misogynistic information they hear online or from their peers. Broader forms of social change, including changes to how social media companies operate, are also crucial. In principle, social media companies and video sharing platforms have policies to discourage extremist content on their sites. For example, individual influencers can be demonetized or even banned [23]. While misogynistic influencers are sometimes censured in this way, enormous quantities of hateful content remains available online and the reach of this content is often increased through recommender algorithms [24]. Stricter regulation is crucial.

## Conclusion

Taken together, our data offer important insights into the ways in which engagement with the manosphere appears to be influencing the behaviour and experiences of children in schools. Teachers report that male pupils discuss misogynistic influencers with some regularly and that misogynistic influencers appear to motivate discriminatory behaviour towards female peers and female teachers. It is clear from this work, and from the broader literature, that the influence of the manosphere over young people needs to be a crucial priority for policy makers, educators and academics.
